# Supplementary material for: Antibiotic de-escalation patterns and outcomes in critically ill patients with suspected pneumonia as informed by bronchoalveolar lavage results
Source: Eur J Clin Microbiol Infect Dis. Author manuscript; Available in PMC 2025 Aug 6. (PMC12321642; doi:10.1007/s10096-025-05144-2)

Supplement

Supplemental Table 1. Summary of resistant pathogens as detected by either multiplex PCR mutation or in culture name and their frequencies. Pathogens were identified based on the presence of specific resistance genes or mechanisms. For example, ['meca/c and mrej', 'mrsa'] indicates that a mecA/C and MREJ mutation were recovered on the Biofire panel, and the culture grew out MRSA.

Abbreviations: CTX-M (cefotaximas-Munich enzyme causing EXBL phenotype), ESBL (Extended-spectrum β-lactamase), mecA/C (Methicillin resistance genes), MREJ (Methicillin resistance gene), MRSA (Methicillin-resistant Staphylococcus aureus), vanc (Vancomycin resistance), beta-L (Beta-lactamase), KPC (Klebsiella pneumoniae carbapenemase), and NDM (New Delhi metallo-β-lactamase).

Resistance as identified by [pcr, culture]	Count
['ctx-m', 'esbl']	24
['meca/c and mrej', 'mrsa']	22
['esbl']	16
['vanc']	10
['ctx-m']	9
['mrsa']	6
['meca/c and mrej']	6
['beta-l']	3
['meca/c and mrej', 'mrsa', 'vanc']	2
['esbl, kpc']	1
['kpc', 'esbl']	1
['meca/c and mrej', 'esbl']	1
['ctx-m', 'esbl', 'vanc']	1
['kpc']	1

['ctx-m', 'ndm']	1

Supplemental Table 2. Frequency of bacterial pathogens in the SCRIPT cohort.

Pathogen	Frequency
Staphylococcus aureus	121
Pseudomonas aeruginosa	89
Viridans streptococcus	49
Escherichia coli	47
Yeast, not Cryptococcus species	46
Staphylococcus coagulase negative	45
Enterococcus faecalis	41
Klebsiella pneumoniae	40
Corynebacterium species	36
Klebsiella aerogenes	21
Haemophilus influenzae	20
Enterobacter aerogenes	18
Enterobacter cloacae complex	18
Serratia marcescens	17
Enterobacter cloacae	16
Enterococcus faecium	16
Klebsiella oxytoca	11
Stenotrophomonas maltophilia	11
Streptococcus agalactiae	11
Citrobacter koseri	10
Lactobacillus species	8
Neisseria species	8

Streptococcus pneumoniae	7
Acinetobacter calcoaceticus-baumannii complex	6
Beta hemolytic streptococci, group f	6
Proteus spp.	6
Acinetobacter baumannii	5
Beta hemolytic streptococci, group c	5
Burkholderia cepacia complex	5
Stomatococcus species	5
Achromobacter xylosoxidans	4
Citrobacter freundii	4
Beta hemolytic streptococci, group g	3
Hafnia alvei	3
Streptococcus pseudopneumoniae	3
Candida albicans	2
Candida tropicalis	2
Chryseobacterium indologenes	2
Elizabethkingia meningoseptica	2
Enterococcus avium	2
Granulicatella adiacens	2
Legionalla pneumophilia	2
Moraxella catarrhalis	2
Achromobacter denitrificans	1
Achromobacter species	1
Acinetobacter ursingii	1
Actinomyces odontolyticus	1

Arcanobacterium haemolyticum	1
Beta-hemolytic streptococci, not group a, b, c, d, f, or g	1
Burkholderia gladioli	1
Candida auris	1
Candida glabrata	1
Chryseobacterium gleum	1
Enterococcus raffinosus	1
Enterococcus species	1
Granulicatella species	1
Morganella morganii	1
Neisseria meningitidis	1
Providencia stuartii	1
Pseudomonas fluorescens	1
Raoultella ornithinolytica	1
Streptococcus constellatus	1
Streptococcus mitis oralis	1
Streptococcus pyogenes	1

Supplemental Table 3. Breakdown of pneumonia categories and etiologies.

Etiology	Category	count
**Bacterial**	CAP	48
	HAP	83
	VAP	157
Bacterial/viral	CAP	19
	HAP	34
	VAP	145
Microbiology-negative	CAP	34
	HAP	72
	VAP	45
Viral	CAP	49
	HAP	68
	VAP	59
Non-pneumonia control	NPC	114
**Total**		927

Supplemental Table 4. Frequency of Viral Pathogens in the SCRIPT Cohort.

Virus	Freq
**SARS-Cov-2**	303
Influenza	18
Human Rhinovirus/Enterovirus	17
Coronavirus (not SARS-Cov-2)	13
Parainfluenza	9
Adenovirus	8
Respiratory Syncytial Virus	8
Human Metapneumovirus	5
CMV	3
HSV	3
Herpes Zoster	2

Supplemental Table 5. Clinical outcomes, by sex, with p-values in the addended text

	Resistant		Susceptible		Viral		Bacterial/viral		Microbiology-negative		Non-pneumonia control		Overall	
	Female	Male	Female	Male	Female	Male	Female	Male	Female	Male	Female	Male	Female	Male
	**(N=13)**	**(N=28)**	**(N=43)**	**(N=80)**	**(N=43)**	**(N=50)**	**(N=28)**	**(N=48)**	**(N=54)**	**(N=53)**	**(N=48)**	**(N=46)**	**(N=229)**	**(N=305)**
**Unfavorable Outcomes**	5 (38.5%)	14 (50.0%)	20 (46.5%)	30 (37.5%)	19 (44.2%)	22 (44.0%)	12 (42.9%)	23 (47.9%)	24 (44.4%)	21 (39.6%)	21 (43.8%)	22 (47.8%)	101 (44.1%)	132 (43.3%)
**Duration of ICU Stay (Median [Q1, Q3])**	16.0 [8.0, 20.0]	13.5 [8.0, 18.0]	10.0 [7.0, 21.0]	10.0 [6.0, 18.0]	14.0 [10.0, 20.5]	15.0 [9.2, 20.0]	15.0 [10.0, 25.5]	18.0 [12.0, 32.0]	10.0 [6.0, 16.0]	11.0 [7.0, 21.0]	8.5 [5.0, 17.2]	8.5 [5.0, 16.8]	11.0 [7.0, 20.0]	12.0 [7.0, 20.0]
**Duration of Intubation (Median [Q1, Q3])**	12.0 [4.0, 16.0]	9.5 [6.0, 15.0]	8.0 [5.0, 13.5]	7.0 [4.8, 13.0]	11.0 [6.0, 17.0]	11.5 [7.0, 17.5]	13.0 [6.0, 23.8]	13.0 [6.0, 26.2]	7.5 [5.0, 12.8]	10.0 [4.0, 18.0]	5.5 [3.0, 11.2]	6.0 [3.0, 11.8]	9.0 [4.0, 14.0]	9.0 [5.0, 16.0]

--- Resistant Bacterial Pneumonia ---
Fisher's Exact Test for Unfavorable Outcomes: p = 0.5240
Mann-Whitney U Test for ICU Days: p = 0.9218
Mann-Whitney U Test for Intubation Days: p = 0.9664

--- Susceptible Bacterial Pneumonia ---
Fisher's Exact Test for Unfavorable Outcomes: p = 0.3434
Mann-Whitney U Test for ICU Days: p = 0.8545
Mann-Whitney U Test for Intubation Days: p = 0.6606

--- Microbiology-negative ---
Fisher's Exact Test for Unfavorable Outcomes: p = 0.6965
Mann-Whitney U Test for ICU Days: p = 0.4192
Mann-Whitney U Test for Intubation Days: p = 0.6065

--- Non-Pneumonia Control ---
Fisher's Exact Test for Unfavorable Outcomes: p = 0.8361
Mann-Whitney U Test for ICU Days: p = 0.9276

Mann-Whitney U Test for Intubation Days: p = 0.9003

--- Viral ---
Fisher's Exact Test for Unfavorable Outcomes: p = 1.0000
Mann-Whitney U Test for ICU Days: p = 0.7343
Mann-Whitney U Test for Intubation Days: p = 0.9446

--- Bacterial/viral ---
Fisher's Exact Test for Unfavorable Outcomes: p = 0.8120
Mann-Whitney U Test for ICU Days: p = 0.2212
Mann-Whitney U Test for Intubation Days: p = 0.7668

Supplemental Figure 1. Average NAT score of days 1-7 relative to BAL collection, stratified by sex

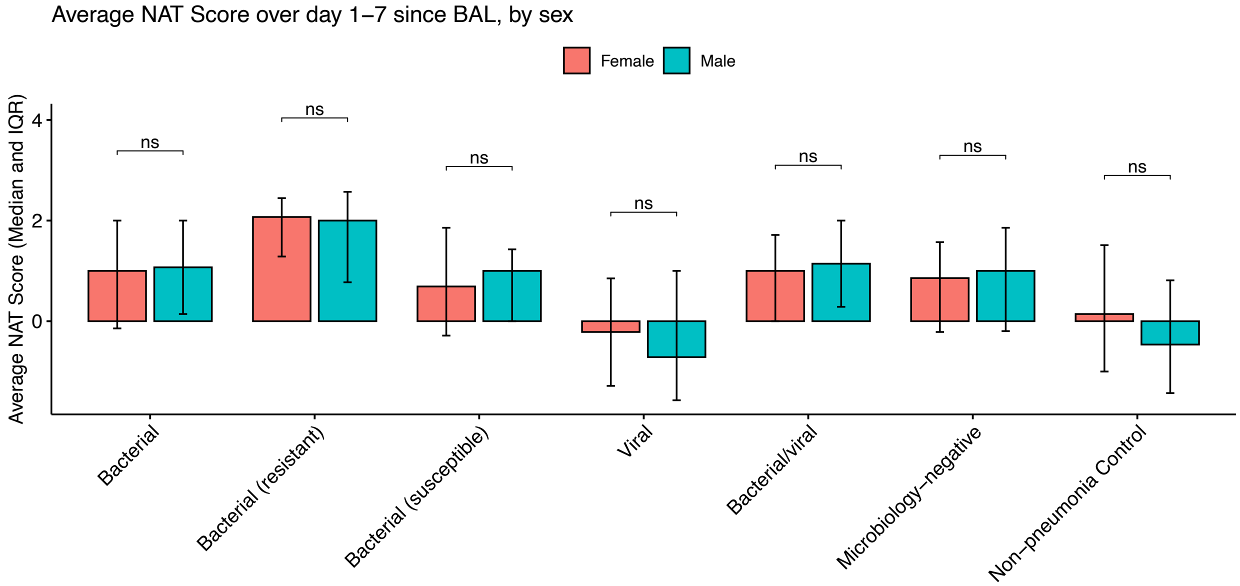

Supplement: Supplemental [file NIHMS2085937-supplement-Supplemental.pdf]
