# Supplementary material for: Antibiotic de-escalation patterns and outcomes in critically ill patients with suspected pneumonia as informed by bronchoalveolar lavage results
Source: Eur J Clin Microbiol Infect Dis. 2025 May 3;44(8):1861–71. doi: 10.1007/s10096-025-05144-2 (PMC12321642; doi:10.1007/s10096-025-05144-2)
Supplement: Supplementary file 1 [file 10096_2025_5144_MOESM1_ESM.docx]

Supplement

**Supplemental Table 1.** Summary of resistant pathogens as detected by either multiplex PCR mutation or in culture name and their frequencies. Pathogens were identified based on the presence of specific resistance genes or mechanisms. For example, ['meca/c and mrej', 'mrsa'] indicates that a mecA/C and MREJ mutation were recovered on the Biofire panel, and the culture grew out MRSA. 

Abbreviations: CTX-M (cefotaximas-Munich enzyme causing EXBL phenotype), ESBL (Extended-spectrum β-lactamase), mecA/C (Methicillin resistance genes), MREJ (Methicillin resistance gene), MRSA (Methicillin-resistant Staphylococcus aureus), vanc (Vancomycin resistance), beta-L (Beta-lactamase), KPC (Klebsiella pneumoniae carbapenemase), and NDM (New Delhi metallo-β-lactamase). 

| Resistance as identified by [pcr, culture] | Count |
| --- | --- |
| ['ctx-m', 'esbl'] | 24 |
| ['meca/c and mrej', 'mrsa'] | 22 |
| ['esbl'] | 16 |
| ['vanc'] | 10 |
| ['ctx-m'] | 9 |
| ['mrsa'] | 6 |
| ['meca/c and mrej'] | 6 |
| ['beta-l'] | 3 |
| ['meca/c and mrej', 'mrsa', 'vanc'] | 2 |
| ['esbl, kpc'] | 1 |
| ['kpc', 'esbl'] | 1 |
| ['meca/c and mrej', 'esbl'] | 1 |
| ['ctx-m', 'esbl', 'vanc'] | 1 |
| ['kpc'] | 1 |
| ['ctx-m', 'ndm'] | 1 |

**Supplemental Table 2.** Frequency of bacterial pathogens in the SCRIPT cohort.

| Pathogen | Frequency |
| --- | --- |
| *Staphylococcus aureus* | 121 |
| *Pseudomonas aeruginosa* | 89 |
| *Viridans streptococcus* | 49 |
| *Escherichia coli* | 47 |
| *Yeast, not Cryptococcus species* | 46 |
| *Staphylococcus coagulase negative* | 45 |
| *Enterococcus faecalis* | 41 |
| *Klebsiella pneumoniae* | 40 |
| *Corynebacterium species* | 36 |
| *Klebsiella aerogenes* | 21 |
| *Haemophilus influenzae* | 20 |
| *Enterobacter aerogenes* | 18 |
| *Enterobacter cloacae complex* | 18 |
| *Serratia marcescens* | 17 |
| *Enterobacter cloacae* | 16 |
| *Enterococcus faecium* | 16 |
| *Klebsiella oxytoca* | 11 |
| *Stenotrophomonas maltophilia* | 11 |
| *Streptococcus agalactiae* | 11 |
| *Citrobacter koseri* | 10 |
| *Lactobacillus species* | 8 |
| *Neisseria species* | 8 |
| *Streptococcus pneumoniae* | 7 |
| *Acinetobacter calcoaceticus-baumannii complex* | 6 |
| *Beta hemolytic streptococci, group f* | 6 |
| *Proteus spp.* | 6 |
| *Acinetobacter baumannii* | 5 |
| *Beta hemolytic streptococci, group c* | 5 |
| *Burkholderia cepacia complex* | 5 |
| *Stomatococcus species* | 5 |
| *Achromobacter xylosoxidans* | 4 |
| *Citrobacter freundii* | 4 |
| *Beta hemolytic streptococci, group g* | 3 |
| *Hafnia alvei* | 3 |
| *Streptococcus pseudopneumoniae* | 3 |
| *Candida albicans* | 2 |
| *Candida tropicalis* | 2 |
| *Chryseobacterium indologenes* | 2 |
| *Elizabethkingia meningoseptica* | 2 |
| *Enterococcus avium* | 2 |
| *Granulicatella adiacens* | 2 |
| *Legionalla pneumophilia* | 2 |
| *Moraxella catarrhalis* | 2 |
| *Achromobacter denitrificans* | 1 |
| *Achromobacter species* | 1 |
| *Acinetobacter ursingii* | 1 |
| *Actinomyces odontolyticus* | 1 |
| *Arcanobacterium haemolyticum* | 1 |
| *Beta-hemolytic streptococci, not group a, b, c, d, f, or g* | 1 |
| *Burkholderia gladioli* | 1 |
| *Candida auris* | 1 |
| *Candida glabrata* | 1 |
| *Chryseobacterium gleum* | 1 |
| *Enterococcus raffinosus* | 1 |
| *Enterococcus species* | 1 |
| *Granulicatella species* | 1 |
| *Morganella morganii* | 1 |
| *Neisseria meningitidis* | 1 |
| *Providencia stuartii* | 1 |
| *Pseudomonas fluorescens* | 1 |
| *Raoultella ornithinolytica* | 1 |
| *Streptococcus constellatus* | 1 |
| *Streptococcus mitis oralis* | 1 |
| *Streptococcus pyogenes* | 1 |

**Supplemental Table 3.** Breakdown of pneumonia categories and etiologies. 

| Etiology | Category | count |
| --- | --- | --- |
| Bacterial | CAP | 48 |
|  | HAP | 83 |
|  | VAP | 157 |
| Bacterial/viral | CAP | 19 |
|  | HAP | 34 |
|  | VAP | 145 |
| Microbiology-negative | CAP | 34 |
|  | HAP | 72 |
|  | VAP | 45 |
| Viral | CAP | 49 |
|  | HAP | 68 |
|  | VAP | 59 |
| Non-pneumonia control | NPC | 114 |
| Total |  | 927 |

**Supplemental Table 4.** Frequency of Viral Pathogens in the SCRIPT Cohort.

| Virus | Freq |
| --- | --- |
| SARS-Cov-2 | 303 |
| Influenza | 18 |
| Human Rhinovirus/Enterovirus | 17 |
| Coronavirus (not SARS-Cov-2) | 13 |
| Parainfluenza | 9 |
| Adenovirus | 8 |
| Respiratory Syncytial Virus | 8 |
| Human Metapneumovirus | 5 |
| CMV | 3 |
| HSV | 3 |
| Herpes Zoster | 2 |

**Supplemental Table 5.** Clinical outcomes, by sex, with p-values in the addended text

|  | Resistant |  | Susceptible |  | Viral |  | Bacterial/viral |  | Microbiology-negative |  | Non-pneumonia control |  | Overall |  |
| --- | --- | --- | --- | --- | --- | --- | --- | --- | --- | --- | --- | --- | --- | --- |
|  | Female | Male | Female | Male | Female | Male | Female | Male | Female | Male | Female | Male | Female | Male |
|  | **(N=13)** | **(N=28)** | **(N=43)** | **(N=80)** | **(N=43)** | **(N=50)** | **(N=28)** | **(N=48)** | **(N=54)** | **(N=53)** | **(N=48)** | **(N=46)** | **(N=229)** | **(N=305)** |
| Unfavorable Outcomes | 5 (38.5%) | 14 (50.0%) | 20 (46.5%) | 30 (37.5%) | 19 (44.2%) | 22 (44.0%) | 12 (42.9%) | 23 (47.9%) | 24 (44.4%) | 21 (39.6%) | 21 (43.8%) | 22 (47.8%) | 101 (44.1%) | 132 (43.3%) |
| Duration of ICU Stay  (Median [Q1, Q3]) | 16.0 [8.0, 20.0] | 13.5 [8.0, 18.0] | 10.0 [7.0, 21.0] | 10.0 [6.0, 18.0] | 14.0 [10.0, 20.5] | 15.0 [9.2,  20.0] | 15.0 [10.0, 25.5] | 18.0 [12.0, 32.0] | 10.0 [6.0, 16.0] | 11.0 [7.0, 21.0] | 8.5 [5.0, 17.2] | 8.5 [5.0, 16.8] | 11.0 [7.0, 20.0] | 12.0 [7.0, 20.0] |
| Duration of Intubation (Median [Q1, Q3]) | 12.0 [4.0, 16.0] | 9.5 [6.0, 15.0] | 8.0 [5.0, 13.5] | 7.0 [4.8, 13.0] | 11.0 [6.0, 17.0] | 11.5 [7.0, 17.5] | 13.0 [6.0, 23.8] | 13.0 [6.0, 26.2] | 7.5 [5.0, 12.8] | 10.0 [4.0, 18.0] | 5.5 [3.0, 11.2] | 6.0 [3.0, 11.8] | 9.0 [4.0, 14.0] | 9.0 [5.0, 16.0] |

--- Resistant Bacterial Pneumonia ---

Fisher's Exact Test for Unfavorable Outcomes: p = 0.5240

Mann-Whitney U Test for ICU Days: p = 0.9218

Mann-Whitney U Test for Intubation Days: p = 0.9664

--- Susceptible Bacterial Pneumonia ---

Fisher's Exact Test for Unfavorable Outcomes: p = 0.3434

Mann-Whitney U Test for ICU Days: p = 0.8545

Mann-Whitney U Test for Intubation Days: p = 0.6606

--- Microbiology-negative ---

Fisher's Exact Test for Unfavorable Outcomes: p = 0.6965

Mann-Whitney U Test for ICU Days: p = 0.4192

Mann-Whitney U Test for Intubation Days: p = 0.6065

--- Non-Pneumonia Control ---

Fisher's Exact Test for Unfavorable Outcomes: p = 0.8361

Mann-Whitney U Test for ICU Days: p = 0.9276

Mann-Whitney U Test for Intubation Days: p = 0.9003

--- Viral ---

Fisher's Exact Test for Unfavorable Outcomes: p = 1.0000

Mann-Whitney U Test for ICU Days: p = 0.7343

Mann-Whitney U Test for Intubation Days: p = 0.9446

--- Bacterial/viral ---

Fisher's Exact Test for Unfavorable Outcomes: p = 0.8120

Mann-Whitney U Test for ICU Days: p = 0.2212

Mann-Whitney U Test for Intubation Days: p = 0.7668

**Supplemental Figure 1.** Average NAT score of days 1-7 relative to BAL collection, stratified by sex
